# Outcomes of kidneys used for transplantation: an analysis of survival and function

**DOI:** 10.3389/frtra.2024.1335999

**Published:** 2024-03-05

**Authors:** Timothy L. Pruett, Paola Martin, Diwakar Gupta

**Affiliations:** ^1^ Division of Transplantation, University of Minnesota School of Medicine, Minneapolis, MN, United States; ^2^ ODT, Kelley School of Business, Indiana University, Bloomington, IN, United States; ^3^ IROM, The McCombs School of Business at University of Texas (Austin), Austin, TX, United States

**Keywords:** kidney transplantation, outcomes, live donation, deceased donation, eGFR

## Abstract

**Introduction:**

Kidney transplant recipients expect to survive the procedure with sufficient renal function for reliable dialysis freedom.

**Methods:**

Transplant outcomes (survival and estimated renal function) were assessed after live and deceased donor transplantation from the US national database. Outcomes were stratified by age (donor and recipient) and donor type.

**Results:**

Aggregate recipient outcomes were better transplanting living vs deceased donated kidneys. However, when stratified by the one-year renal function (within KDIGO CKD stage stratifications), surviving recipients had clinically similar dialysis-freedom, irrespective of donor type or age. The major outcome differences for recipients of age-stratified live and deceased kidneys was 1) the increasing frequency of one-year graft failures and 2) the increasing likelihood of severely limited renal function (CKD 4/5) with advancing donor age. Over 30% of recipients of deceased kidneys >65 years had either one-year graft failure or severely limited renal function contrasted to less than 15% of recipients of live kidneys aged >65 years.

**Conclusions:**

Evolving techniques to reduce adverse events after urgent vs elective procedures, plus improved transplant outcome predictability with increased-age deceased donor kidneys using advanced predictive analytics (using age-stratified live kidney transplantation outcomes as a relevant reference point) should facilitate similar kidney transplant outcomes, irrespective of donor type.

## Background

The demand for kidney transplantation has always exceeded kidney availability, so the non-utilization rate in excess of 25% (https: optn.transplant.hrsa.gov/data) of deceased donor (DD) kidney offers in the United States is vexing (1–3). However, the most frequent reason provided for declining an available kidney is that the offered kidney is of insufficient quality (4). Declining to transplant a kidney that will not benefit a recipient is a good medical decision, and acceptance and transplantation of kidneys should depend upon the likelihood that recipients will benefit. Declines of offered DD kidneys are not uniform; older deceased donor kidneys are more frequently refused than those from younger donors (5). The non-use of donor kidneys increases with the recognized loss of glomerular filtration rate (GFR) that occurs with advancing age (6). This becomes a system issue as older individuals die at a higher frequency than younger people and constitute the most prominent population to expand kidney availability (7). In contrast to the “underutilization” of DD kidneys, live donated kidneys have near 100% utilization, irrespective of donor age. In the US, over 50% of offered kidneys from DD aged >65 years are declined, but over 99.9% of kidneys from living donors (LD) aged >65 years are transplanted (8, 9). Outcome differences between LD and deceased donor kidney transplant (DDKT) therefore provide insight into clinical expectations and use.

The premise for this analysis is that the expected outcome after kidney transplantation is that the recipients will survive the procedure with sufficient renal function to permit durable dialysis-freedom. The uncertainty associated with obtaining that goal is the subject of this analysis. Age-related diseases and co-morbidities decrease the likelihood for recipient survival after operative and immunologic stress. Sufficient recipient renal function is the foundation of successful kidney transplantation, although a precise definition has been fluid. In surviving patients, the 1-year residual renal function has been correlated with 10-year graft function (10) and recipients with estimated glomerular filtration rate (eGFR) in excess of the chronic kidney disease (CKD) 3a range have similar 10-year death-censored graft survival. However, it has also been shown that recipients of kidneys from DD aged >65 years with CKD 4 or less [<estimated glomerular filtration rate at 1 year (eGFR-1) <30 ml/min/m^2^] have a lesser survival than a matched cohort of never-transplanted candidates on the waitlist (11). Bae et al. used the Estimated Post-transplant Survival (EPTS) and Kidney Donor Profile Index (KDPI) to ask the question of which candidates would benefit from a “marginal kidney” transplant (12). While demonstrating that the average “marginal” kidney provided the average older recipient a benefit, the discussion did not address the spectrum of renal function or the relative risk that recipients of these kidneys would fail to gain beneficial renal function.

Live donation is the type of kidney transplant that provides optimal recipient outcomes (13, 14), but it has been reported, using propensity matching, that LD and DD kidney recipient outcomes are clinically similar (15). This suggests a process improvement opportunity, to reduce outcome differences between DD and LD kidney transplantation, such that outcomes of early and durable graft survival are similar. We therefore performed an analysis of the US transplant database and assessed the spectrum of early graft survival and amounts of renal function after 1 year (eGFR-1) from kidney transplantation. The goal was to identify system domains to facilitate the utilization of DD kidneys without significantly increasing the risks for graft failures and maintaining durable benefit. LD outcomes provided a useful benchmark for this aspirational goal.

## Methods

De-identified demographic and outcome data of deceased and living kidney donors and transplant recipients was obtained for adult first-time single-kidney-only transplants performed from 1 January 2005, through 31 December 2014, using the Standard Transplant Analysis and Research (STAR) file obtained from Organ Procurement and Transplantation Network (OPTN)/United Network for Organ Sharing (UNOS) (16) (University of Texas, Austin. IRB Protocol 2018-12-0026) in accordance with the 1964 Declaration of Helsinki. Assessments until 13 December 2019 allowed a complete 5-year follow-up for all KTs unaffected by the COVID-19 pandemic. The kidney waitlist end-of-year demographics for 2004–2014 was provided by UNOS Research, Richmond, VA, USA (Sarah Taranto, UNOS Research, Personal Communication). The 1-year OPTN data of surviving kidneys provided sufficient information to calculate the transplant glomerular filtration rate (eGFR-1) using the chronic kidney disease epidemiology collaboration equation (CKD_EPI) equation (17). While age and eGFR-1 are continuous variables, categorical divisions were used for comparative purposes using the Kidney Disease Improving Global Outcomes (KDIGO) CKD stages (18) (CKD stage: 1 >90; 2, 60–89; 3a, 45–59; 3b, 30–44, and 4/5 <30 mL/min/1.73 m^2^). Younger DD kidneys (the average DD age was 45 years) were pooled as they are routinely accepted for transplantation and contrasted with recipient outcomes from transplanted DD kidneys grouped into increasing 10-year donor age increments up to >65 years. In addition, kidney donor risk index (KDRI)/KDPI (19) was calculated for DD and the kidneys stratified by KDPI <0.5, 0.50–0.74, 0.75–0.89, and >0.9. The average age of DD kidney recipients was 54 years and recipient outcomes were stratified for simplicity as <55 (younger half), 55–64, and >65 years. The trajectory of death-censored graft survival was plotted using the Kaplan–Meier estimate and the log-rank *p*-value calculations. In addition to live kidney donor age stratification, living donor kidneys were classified into ranges of <15, 15–35, 35–60, and >60 based on Living Kidney Donor Profile Index (LKDPI) (20).

Despite renal function, KDRI and age being continuous variables, these data elements were categorized by conventional KDIGO CKD stratifications, clinical convention, and age in decades of life. The *p*-values were obtained upon running unpaired *t*-tests with unequal variances for continuous variables and chi-square tests for categorical variables. All data and statistical analyses were performed using STATA version MP 17.0 (21).

## Results

### Overall kidney transplants, demographics, and results

**Figure 1:**
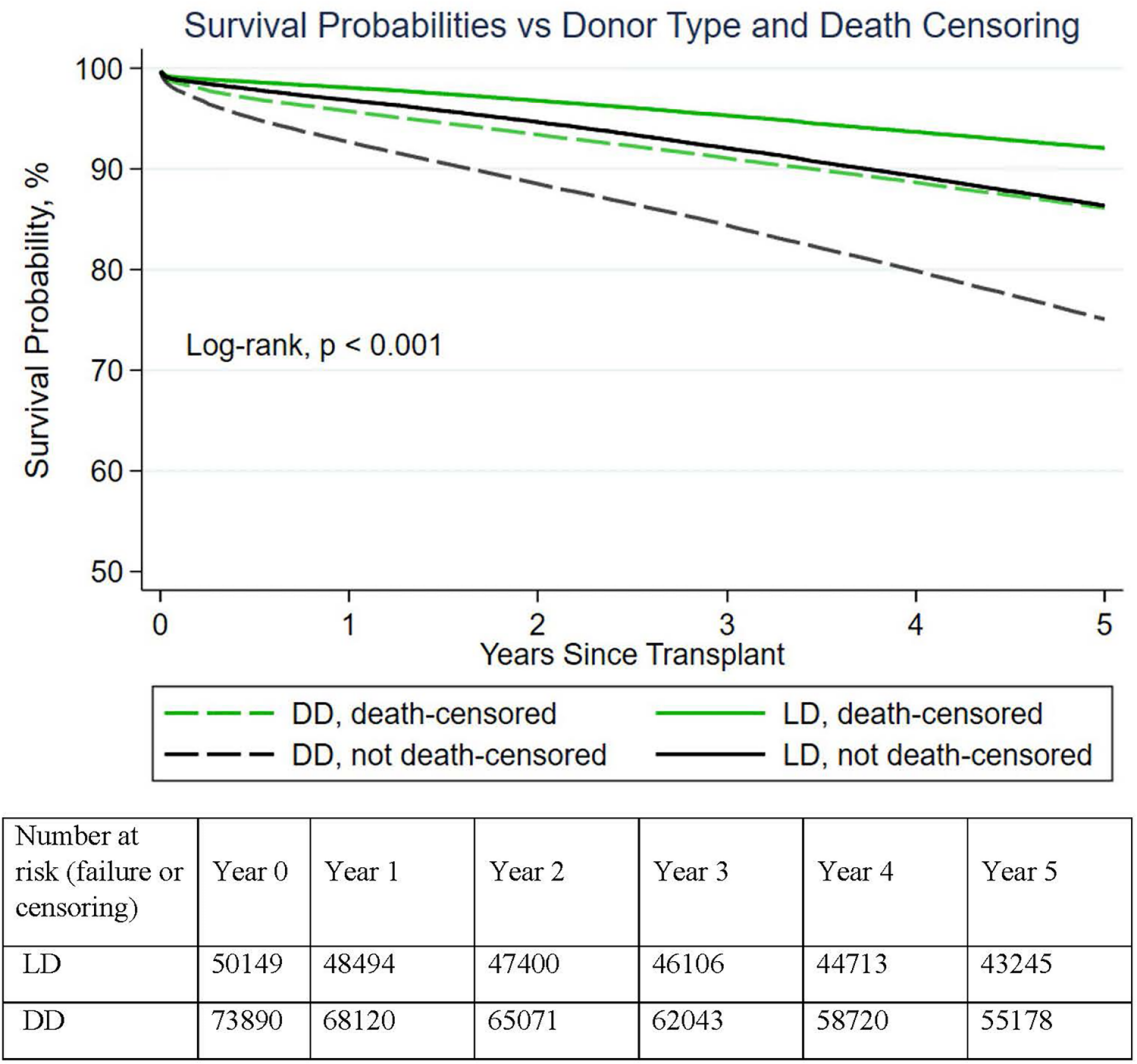
Demonstrated graft survival superiority of live donor vs deceased donor kidneys (with and without death-censoring). In aggregate, LD transplants had significantly higher (death-censored) 1–5-year survival than DD transplants.

Between 2005 and 2014, 124,039 first kidney-only transplants were performed (Table 1), 73,890 (60%) from DD and 50,149 (40%) from LD. Including recipient deaths, 1- and 5-year patient and graft survival of DDKT recipients was 92%/75% and living donor kidney transplant (LDKT) 97%/86%, respectively (*p* < 0.001). Death-censored DD and LDKT 1- and 5-year graft survival was 96%/86% and 98/92%, respectively (*p* < 0.001) (Figure 1). The average DD recipient was older than the LD recipient (54 vs. 48 years). However, DD/LDKT recipients within age groups <45, 45–54, 55–64, or >65 years were 23%/38%, 25%/25%, 34%/24%, and 22%/13%, respectively. LDs were younger than DDs, with the percentage of DDs/LDs aged <45, 45–54, 55–64, or >65 years being 52%/58%, 27%/27%, 17%/13%, and 4/2%, respectively.

**Table 1:** DD and LD kidney donor and recipient characteristics stratified by donor age (*N* = 124,039).

Donor age range	DDKT: 73,890				LDKT: 50,149			
	18–44	45–54	55–64	>65	18–44	45–54	55–64	>65
Not utilized (%)	8	21	34	57	NA	NA	NA	NA
Sample size (*n*)	38,163	20,166	12,581	2,980	28,909	13,725	6,582	933
Donor characteristics								
Mean age [median (IQR^a^)]	31 (23–39), 31	49 (47–52), 49.5	58 (56–61), 58.7	67 (66–69), 68.1	34 (28–40), 33.5	49 (47–52), 49.2	58 (56–60), 58.5	67 (66–68), 67.4
Mean BMI [median (IQR)]	26.4 (23.1–30.2), 27.7	27.6 (24.1–32.1), 28.7	27.6 (24.4–32), 28.6	26.7 (24–30.2), 27.7	26.6 (23.6–29.9), 26.9	26.8 (24.1–29.7), 27	26.5 (23.9–29.3), 26.7	26.3 (23.8–29.2), 26.5
Female (%)	34.5	45.5	46.9	51.4	57.9	65.6	65.7	63.2
Caucasian (%)	66.8	71.6	74	76.9	60.5	76.7	83.4	87
African American (%)	13.8	13.3	10.6	8.7	16	9.2	5.6	3.1
Hispanic (%)	15.6	11.4	11.2	9.5	17.8	10.1	7	5.1
Asian (%)	1.9	2.6	3.1	4.1	4.1	3	3.2	4
Other race (%)	1.9	1.1	1.1	0.8	1.6	1	0.8	0.8
Recipient characteristics								
Mean age [median (IQR)]	52 (42–61), 51.1	56 (47–64), 54.5	61 (53–67), 59.2	65 (59–70), 64.3	46 (36–58), 46.4	51 (43–57), 49.3	57 (47–62), 53.2	66 (58–69), 61.6
Mean BMI [median (IQR)]	28.1 (24.4–32.2), 28.5	28.1 (24.6–32.2), 28.6	28.1 (24.7–31.9), 28.5	27.8 (24.5–31.6), 28.2	27.3 (23.7–31.5), 27.8	27.3 (23.7–31.4), 27.8	27.4 (23.9–31.3), 27.8	27.5 (24.1–31.3), 28
Female (%)	39.5	38.4	37	36.9	38.5	37.5	37.2	37.1
Caucasian (%)	41.9	42.8	44.4	46.5	58.2	72.8	78.2	81.7
African American (%)	34.4	34.5	31.6	29.8	17.8	11.5	8.4	5
Hispanic (%)	16.1	14.2	14.1	13.8	17.7	10.4	7.8	6.1
Asian (%)	5.6	6.5	7.7	8	4.8	4	4.5	5.9
Other races (%)	2	2	2.2	1.9	1.5	1.3	1.1	1.3
Transplant characteristics								
With three or fewer HLA mismatches (%)	28.5	24.7	19.3	14.4	56.9	50.9	47.3	39.3
Delayed graft function (%)	23	29.9	30.2	29.5	3.2	3.8	3.9	5.1
Mean cold ischemia time (h) [median (IQR)]	16.4 (11.1–22.4), 17.6	16.5 (11.3–22.6), 17.8	17 (11.8–23), 18.2	17.3 (12.3–23.5), 18.6	1 (0.7–2), 2.9	1 (0.73–2), 2.2	1 (0.7–2), 2.2	1 (0.6–2), 2.3
Biologically related (%)	NA	NA	NA	NA	61	50	43	32
Transplant outcomes								
1-year patient and graft survival (%)	94	92	88	86	97	97	96	94
With eGFR-1 > 90 ml/min (%)	16	6	3	1	15	6	3	2
With eGFR-1 of 60–90 ml/min (%)	47	33	24	15	52	42	31	22
With eGFR-1 of 45–60 ml/min (%)	23	32	31	30	23	35	37	39
With eGFR-1 of 30–45 ml/min (%)	10	23	30	38	8	15	24	30
With eGFR-1 < 30 ml/min (%)	3	7	12	17	2	3	5	8

^a^ IQR, interquartile range.

*Supply/demand for kidney transplantation: demographics of the national waitlist and deceased and live donor kidney recipients*: Between 2005 and 2015, the national waitlist grew from 64,838 to 101,915 candidates. Candidates aged <45 years represented 25%–30% of the WL, candidates aged 50–64 years constituted over 40% of the national waitlist, and candidates aged >65 years had the greatest percentage increase, from 15% (2005) to 25% (2015). The ethnicity of the kidney waitlist shifted slightly from the end of year in 2005 to 2015: Caucasian (Cauc) 40% to 36%; African Americans (AA) 35% to 34%; Hispanic/Latino (H/L) 17% to 20%; Asian (As) 6% to 8%; and Native American/Alaska Native/Pacific Islander/multiracial (Other) 3% to 2%, respectively. The male/female percentage of the WL has been approximately 60%/40%, with a gradual decline in female candidates (Supplementary Figure S1).

**Figure 2:**
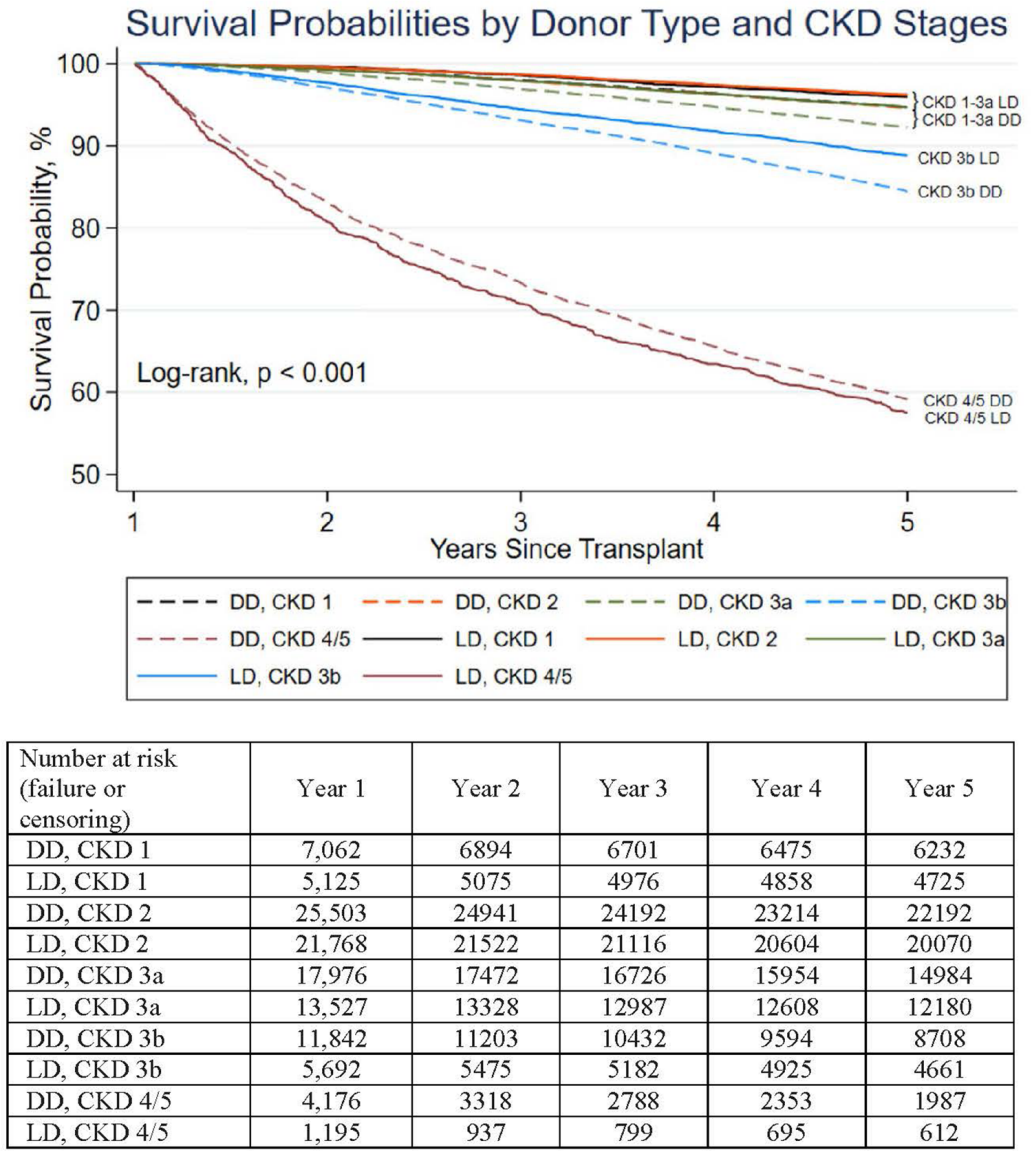
Death-censored DD and LD graft survival of kidneys surviving 1 year stratified by CKD stage 1 year after kidneys transplantation. Stratified by CKD stages, death-censored 1–5 year survival of LD contrasted to DD transplants was clinically similar (although statistically different, *p* < 0.05 for all stages except CKD 4/5). The differences in 5-year death-censored graft survival between LD and DD transplants for CKD 1, 2, 3a, 3b, 4/5 were 1.4, 1.9, 1.8, 6, −1.4%, respectively.

The ethnicity of DDKT recipients differed from candidates on the WL (Δ ethnicity; % DDKT recipient ethnicity − % waitlist ethnicity); DDKT recipients were Cauc 43 (+4%), AA 34 (−1%), H/L 15 (−3%), As 6 (−1%), and Other 2% (0.0). The percentage of women receiving a DDKT decreased as the recipient age increased. The ethnic disparity for LD recipients differed markedly from the waitlist; >60% were Cauc with ethnic disparity from the waitlist >30% and a commensurate reduction for other ethnicities: AA 15% (−20%); H/L 14% (−6%); and As 5% (−4%) (Figure 2). Recipients aged >65 years constituted a lesser percentage of total LD and DDKT contrasted to the waitlist, with 13% versus 22% of transplants performed. However, >80% of LDKT recipients aged >65 years were Caucasian. The kidney donor gender differed: the M/F ratio was approximately 60/40 for DD kidneys and 40/60 for LD kidneys.

### Kidney use, donor age, and KDPI

Of all deceased donor kidneys offered for transplantation, 19.6% were declined. The mean age of the DD was 45 years, and >92% of kidneys offered from DD aged <45 years were transplanted (for kidneys with KDPI < 0.5, 96% of offered kidneys were transplanted). As the DD age (Table 1) or KDPI (Supplementary Table S2) increased, the kidney refusal rate rose incrementally: a donor age of 45–54 years or KDPI of 0.5–0.74 had a refusal rate of approximately 20%; of DD kidneys aged 55–64 years or a KDPI of 0.75–0.89, 34%–38% were refused; and kidneys from DDs aged >65 years or a KDPI > 0.9, >50% of offered kidneys were declined for transplantation. In contrast, <0.1% of retrieved LD kidneys were not transplanted, irrespective of donor age. There was no available OPTN data for measured donor GFR, the GFR threshold required for live donor acceptance, nor the numbers of candidate donors declined for inadequate GFR.

### One-year transplant success (patient and graft survival), by donor and recipient age

#### DDKT

One-year patient and graft survival declined as DD age and KDPI increased (Table 1); recipients of kidneys from DDs aged <45, 45–54, 55–64, and >65 years had a 1-year patient/graft survival of 94%, 92%, 88%, and 86%, respectively. However, the outcomes are confounded by recipient ages; as the DD age advanced from <45 to >65 years, the mean recipient age increased from 51 to 64 years. Recipient 1-year patient/graft survival of kidneys stratified by KDPI was similar. Kidney recipients, with KDPI < 0.5 kidneys, had a 1-year patient/graft survival of 94% (recipient age, 53 years). As KDPI increased to 0.75–0.9 or >0.9, the recipients were older (61 and 64 years) (Supplementary Table S1) and had a lower patient/graft survival, at 87% and 84%, respectively. One-year mortality was uncommon in younger recipients (aged <55 years): <3.5% with any aged DD kidneys (Supplementary Table S1). However, 1-year graft failures increased threefold, from 5% to 15%, as the DD age increased from <45 or >65 years. Recipients aged >65 years had greater 1-year mortality as the DD age increased: 6%–10% when the DD age was <45 or >65 years, respectively (and 1-year graft failures increasing from 8% and 16%, respectively).

#### LDKT

One-year recipient death and graft failure also increased with advancing LD age, but to a lesser degree than DDKT (*p* < 0.001). LD age <45, 45–54, 55–64, or >65 years provided recipient 1-year patient/graft survival of 97%, 97%, 96%, and 94%, respectively (Table 1). As with DD kidneys, older LD kidneys were transplanted predominantly into older recipients. While LDKT and DDKT outcomes were statistically different for all aged donors, the differences became clinically relevant in recipients (mean age, 62 years) of LD kidneys aged >65 years, with a 1-year mortality of 4% and graft failure of 6% (Supplementary Table S1).

*Calculation and stratification of renal function of transplants surviving 1 year:* On account of missing data, eGFR-1 could be calculated for 66,559 (90%) DD and 47,307 (94%) LD recipients.

### Spectrum of post-transplant renal function (eGFR-1)

**Figure 3:**
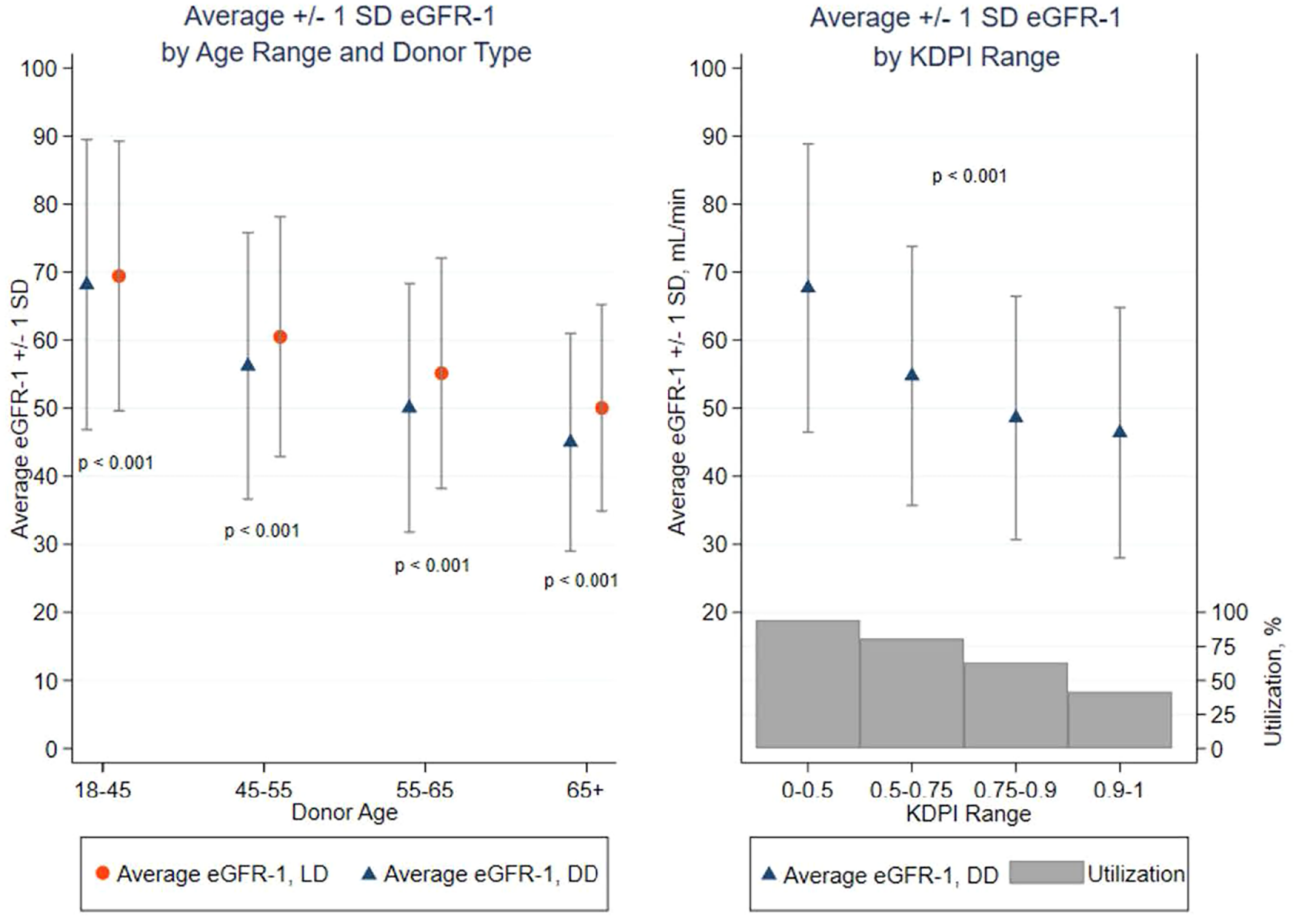
Average eGFR-1 ± 1 standard deviation (SD) by Age range, donor type, and KDPI range: diminution of eGFR-1 with advancing LD and DD age and increasing KDPI. *p* < 0.001 between LD and DD within donor age and *p* < 0.001 for average eGFR-1 by category of LD age and DD age or KDPI. Percent organ offers utilized/transplanted by KDPI range. >99.9% of all LD kidneys were transplanted.

The mean eGFR-1 of DD kidney recipients was lower (61 ± 22 mL/min/1.73 m^2^) than that after LDKT (65 ± 20 ml/min/m^2^; *p* < 0.001). Donor age had a strong association with reduced mean recipient eGFR-1 (*p* < 0.001) (Figure 3). Recipients of DD kidneys aged <45 years had a mean eGFR-1 of 68 ml/min/1.73 m^2^, clinically similar to recipients of LD kidneys aged <45 years (69 ml/min/m^2^). Of DD and LD recipients transplanted with kidneys aged <45 years, 87% and 91%, respectively, had an eGFR-1 > 45 ml/min/1.73 m^2^ (CKD 1–3a range eGFR), with few recipients (3% DD/2% LD) being left with severely reduced eGFR-1 < 30 ml/min/1.73 m^2^. However, as the DD age increased (45–54, 55–64, and >65 years), the mean recipient eGFR-1 decreased (56, 50, and 45 ml/min/1.73 m^2^, respectively), the percentage of recipients with an eGFR-1 > 45 ml/min/1.73 m^2^ decreased (70%, 58%, and 46%, respectively), and those with eGFR-1 < 30 ml/min/1.73 m^2^ increased (7%, 12%, and 16.5%, respectively). KDPI stratification of DD kidneys demonstrated a similar trend (Supplementary Table S2). The percentage of surviving recipients with eGFR-1 < 30 ml/min/1.73 m^2^ after transplantation with KDPI 0.75–0.9 or >0.9 kidneys was 14% and 19%, respectively (Supplementary Table S2). LD recipients of kidneys from donors aged 45–54, 55–64, and >65 years had an eGFR-1 > 45 ml/min/1.73 m^2^ in 82%, 72%, and 62% of recipients, respectively, but only 3%, 5%, and 8%, respectively, had an eGFR-1 < 30 ml/min/1.73 m^2^. The eGFR-1 was similar in recipients of differing ethnicities when stratified by DD or LD age.

### Death-censored 5-year graft survival stratified by CKD stage (eGFR-1)

The 5-year death-censored graft survival correlated with the amount of renal function gained 1 year after transplantation (eGFR-1) (*p* < 0.001), but this was not clinically affected by donor source (DD/LD) nor age. Recipients gaining an eGFR-1 > 45 ml/min/1.73 m^2^ (CKD 1, 2, or 3a) had a clinically similar (5-year) death-censored graft function of over 92%. However, within each CKD stage, LD and DD graft survival differed by <2.5% (Figure 2). Recipients with a CKD 3b range eGFR-1 (30–44 ml/min/1.73 m^2^) had a slightly accelerated and clinically noticeable decreased 5-year graft survival that remained >80% at 5 years (4% graft survival difference favored LD recipients). Those recipients with an eGFR-1 < 30 ml/min/1.73 m^2^ had markedly reduced (death-censored) 5-year graft survival, at <60%, irrespective of the kidney source (DD or LD) or age.

## Discussion

A kidney must provide sufficient renal function to be successful, with the corollary being that insufficient renal function should be avoided (when possible). Process differences between LD and DDKT become highly relevant in the selection of kidneys and provide insights into process improvement opportunities. Renal function of live donated kidneys is measured before donation and the elective nature of the procedure permits elective recipient preparation before surgery/transplantation. Deceased kidneys must be assessed quickly based upon clinical judgment of the available clinical information and then transplanted urgently. This analysis provides a comparison of outcomes based upon these two processes. We demonstrated, as did Kasiske et al. (10) and Yohanna et al. (15), that transplant benefit is proportional to the amount of renal function the kidney provides to the recipient, irrespective of donor type. We also demonstrated, unsurprisingly, that post-transplant renal function decreases with advancing donor age. While the (mean) recipient eGFR-1 for LD or DD recipients of kidneys from young donors (<45 years, the younger half of deceased kidney donors) was very similar (almost 70 ml/min/m^2^), as donor age increased from 45 to >65 years, the mean eGFR-1 of kidney recipients incrementally decreased by 20 ml/min/1.73 m^2^, irrespective of donor type (Figure 3). Recipients of LD kidneys consistently gained a modestly greater, age-stratified mean eGFR-1 than DD kidneys, but only 5 ml/min/1.73 m^2^. However, the frequency of LD recipients with marginal renal function at 1 year (CKD 4/5) was reduced approximately twofold, in contrast with recipients of age-adjusted DD kidneys.

Aggregate LDKT outcomes were unsurprisingly superior to those of DDKTs (Figure 1), yet death-censored survival was clinically similar for 1-year survivors with similar amounts of renal function (Figure 2). The LDKT survival superiority was predominantly due to the following: (1) a two- to threefold improvement in recipient 1-year survival with a functioning graft; and (2) the lesser frequency of severely limited renal function associated with less graft function durability (CKD 4/5). One-year graft survival decreased incrementally as donor age increased, but the observation is confounded by advancing recipient age and age-associated co-morbidities (Table 1). Surgical procedures performed urgently (such as DDKTs) are known to have a greater perioperative morbidity and mortality, particularly in older individuals, in contrast to similar procedures performed electively (LDKTs). Surgical quality initiatives that identify processes to reduce morbidity/mortality in elective or urgently performed procedures (22–25) should be integrated into transplant surgical performance and waitlist management. Unfortunately, it was impossible to discern from OPTN data the impact of elective/urgent process differences compared to the impact of recipients gaining (in)sufficient renal function from older DD kidneys. Still, the twofold reduction in 1-year mortality and graft failure observed between older LD versus DDKT recipients, irrespective of kidney donor age, suggests that elective candidate preparation and provision of a kidney with immediate, sufficient renal function is fundamental to successful transplant outcomes.

The main difference of clinical concern comparing LD and DD outcomes was not the mean age-stratified eGFR, rather the likelihood that DD recipients of older kidneys would fail to gain meaningful (eGFR-1 CKD status 3b or higher) renal function even after a successful transplant. Sufficient 1-year renal function is necessary for survival and quality-of-life benefit. While the CKD 4 threshold may not exactly capture the lower threshold for beneficial transplant renal function, the rapid loss of graft survival and lesser survival observed in older recipients (11) suggests that it is close. Of surviving DD recipients of kidneys aged >65 years, 17% had a residual eGFR-1 < 30 ml/min/1.73m^2^, which, coupled with a 1-year graft failure (that included death) of 14%, suggested that almost one-third of DDKT recipients of highly selected kidneys aged >65 years failed to gain a transplant survival benefit. A similar finding was observed in an Australian cohort of LD and DD kidney recipients (26). Of recipients of donor kidneys aged >60 years, 17% had an eGFR-1 < 30 ml/min/m^2^. This report combined kidneys by donor age and did not discuss the spectrum of renal function obtained by donor type. Sufficient renal function is essential for recipient benefit and transplant success, and the association between lesser renal function and graft failure and mortality is intuitive; it must be balanced by the morbidity and mortality of dialysis. There is no consensus of an acceptable (system) graft failure rate nor a minimally acceptable eGFR-1, but the DD kidney refusal rates suggest a visceral clinical threshold exists. It is likely that the relative risk for transplant failure using older, high KDPI kidneys contributes greatly to the refusal of clinicians’ acceptance of these kidneys. Even with a mean benefit greater than remaining on dialysis, the increased likelihood of failure muddles the decision.

Survival expectations have been systematically modified (risk adjustments) to accommodate the observed survival reduction using DD kidneys with advancing age; Extended Criteria Donor (27) and the KDPI (19) are heavily age dependent. The KDPI added co-morbidities, but the robustness of the Index has been lessened after the introduction of successful anti-Hepatitis C Virus (HCV) therapies and race-based modifications. However, no stratification system has been predictive for the spectrum of renal function after transplanting older DD kidneys. This is problematic as sufficient renal function at 1 year (eGFR-1) is associated with transplant success (10, 26). For the CKD 1–3a range of eGFR-1 (>45 ml/min/m^2^), recipients had clinically similar (death-censored 5-year) graft survival (92%–96%), with a difference <2.5% between LD/DD recipients within each CKD stage (Figure 2). With renal function of CKD 3b or lower, the beginning of an inflection point for decreased recipient rate of graft survival occurred, but still remained above 80%. However, when eGFR-1 fell to <30 ml/min/1.73 m^2^, graft survival was markedly reduced. If eGFR-1 could be more reliably predicted, it would impact candidates’ informed consent, decision-making of kidney acceptance by providers, and policy generation and system performance oversight.

Donor age is the major factor associated with reliable renal function after transplantation. Donors aged <45 years provided LD and DD recipients almost 70 ml/min/1.73 m^2^, with <3% of surviving recipients having an eGFR-1 < 30 ml/min/1.73 m^2^. However, with advancing donor age, the mean eGFR-1 decreases and the likelihood for severely limited renal function increases (with diminishing aggregate transplant benefit). Clinical judgment has a limited capacity to rapidly process the plethora of DD clinical data available and determine kidney acceptability/potential function. Even after rejecting 40%–60% of available kidneys, 13% and 16% of recipients (respectively) of kidneys from DDs aged 55–64 or >65 years had an eGFR-1 < 30 ml/min/m^2^. Recipients of kidneys from donors with a KDPI >0.75 had an even greater percentage of CKD 4/5 eGFR-1 (Supplementary Table S2). There is a need to reduce the frequency of recipients gaining marginal renal function after transplantation (older DD kidneys). Advanced predictive analytics holds a promise that with additional critical variables, eGFR-1 after DDKT may become similar to that observed after LDKT. Lasserre et al. applied machine-learning modeling algorithms to pre-donation data of 707 single-center kidney transplants (Eurotransplant and transplant center recipient data) to predict GFR-1 (28). While donor age was the feature with the most predictive power, it was insufficient as a univariate feature to reliably predict eGFR-1. Their data and numbers did not provide sufficient accuracy for clinical use, but the authors saw promise with variable refinements. Martin et al. applied machine-learning algorithms to OPTN data to predict eGFR-1 from >10,000 recipients with reported graft survival with DD kidneys aged >55 years (29). The predictive algorithm learned from the successful DDKTs and then applied the algorithm to matched (mostly by KDRI features) but un-transplanted kidneys to candidates who had been offered the refused kidneys (40% of total offered kidneys). Almost two-thirds of non-utilized (discarded) older DD kidneys had been offered to at least one candidate on the match run with a >80% likelihood of obtaining a post-transplant eGFR-1 > 30 ml/min. The reports are both suggestive that machine-learning algorithms (artificial intelligence) applied to pre-transplant data may predict the likelihood of gaining eGFR-1, possibly similar to that observed after LDKT. There is additional data beyond KDPI variables available within the OPTN database, including renal volume/mass (30, 31), donor biopsy (32, 33), (hypo)perfusion parameters (34), pump (35), and ischemia characteristics (36), that may improve the predictive capacity for post-transplant renal function.

This analysis provides a broad summary of >100,000 kidney transplant outcomes using the data from the OPTN database. The association between graft failure and eGFR-1 trajectories is admittedly oversimplified, as Raynaud et al. (37) demonstrated by the heterogeneity of graft loss patterns in recipients with the inclusion of post-transplant variables. However, the decision to transplant a kidney is made from the plethora of data available before transplantation, with the expectation that the recipient will survive the operation with sufficient renal function. The main purpose of this analysis was to contrast patient and graft survival with the spectrum of eGFR-1 after LD and DDKT (stratified by age). Despite the significant demographic differences of recipients and donors of live and deceased kidneys, (death-censored) graft survival was remarkably similar with renal function stratification. The findings by Kasiske et al. (10), Lim et al. (26) (eGFR-1 stratified), and Yohanna et al. (15) (propensity matched LD/DD) provided conclusions similar to this analysis. However, none demonstrated the eGFR-1 spectrum stratified by donor age and type. The demographic differences of ethnicity, age, immunologic mismatch, biological relationship, delayed graft function, hemodynamic and cold and warm ischemia times are all relevant to transplant outcomes, but after 1 year appear to become less important when contrasted to residual renal function. This report is a population conclusion and not reflective of individual outcomes. However, informed consent, policy, and systems decisions about kidney utilization require this type of information and kidneys unlikely to provide sufficient renal function to benefit a recipient should not be transplanted.

System-wise, the benefit of kidney transplantation is proportional to the amount of renal function provided to the recipient, irrespective of organ source or donor age. Aspirational system goals should be to eliminate the outcome differences between LDKTs and DDKTs. While the causes of death with a functioning graft have been characterized (38), the contribution of insufficient renal function during the first year upon early mortality/graft loss remains to be better characterized. More attention and better prediction of amounts of recipient renal function required to overcome peri-transplant stress is required. Meta-analyses of LDKT outcomes utilizing donor and recipient characteristics confirmed the importance of donor age (and the absence of ethnicity influence). While LD eGFR-1 was not available, kidney size/gender mismatch was a surrogate of available renal function, and correlated with outcomes (39, 40). In our study, 42% of recipients of DD kidneys aged >65 years gained an eGFR-1 > 45 ml/min/1.73 m^2^ with excellent potential for 10-year survival; but 15% failed to survive to 1 year and 17% of survivors had severely limited eGFR, diminishing recipient benefit. In contrast, recipients of similarly aged LD kidneys had only 8% graft failures at 1 year and 63% of recipients had an eGFR-1 > 45 ml/min/1.73 m^2^ (and only 8% < 30 ml/min/1.73 m^2^). Reducing 1-year death and graft failure of DDKT recipients while improving the predictive capacity to avoid limited renal function is required to improve effective kidney use. The goal of kidney transplantation should be to provide reliable, predictable outcomes irrespective of kidney source or donor age.

## Data Availability

Publicly available datasets were analyzed in this study. These data can be found here: Standard Transplant Analysis and Research file: https://optn.transplant.hrsa.gov/data/view-data-reports/request-data/data-request-instructions/.
