# Supplementary material for: Outcomes of kidneys used for transplantation: an analysis of survival and function
Source: Front Transplant. 2024 Mar 5;3:1335999. doi: 10.3389/frtra.2024.1335999 (PMC11235350; doi:10.3389/frtra.2024.1335999)

Supplemental Tables

1. **DD and LDKT patient/graft survival and eGFR-1 spectrum by donor age and recipient age, with 3 categories for recipient age**……………………………………………………………………………………………….S 1
2. **Donor and Recipient characteristics, stratified by KDPI and LKDPI**……………………………………….S 2

Supplemental Table 1. Outcomes and eGFR-1 spectrum, stratified by DD and LD donor and recipient age within 3 categories for recipient age: demonstration of increasing risk of recipient death/graft failure with advancing age and lesser eGFR-1

		DDKT: 73,890				LDKT: 50,149			
		D Age: 18-45	D Age: 45-55	D Age: 55-65	D Age: 65+	D Age: 18-45	D Age: 45-55	D Age: 55-65	D Age: 65+
% 1-year patient survival (*n*)	R Age: 18-55	98.1 (*21,260*)	97.5 (*9,290*)	96.7 (*3,661*)	96.5 (*368*)	99.1 (*19,379*)	99.1 (*9,027*)	99.4 (*2,691*)	97.0 (*203*)
	R Age: 55-65	96.3 (*10,865*)	94.9 (*6,412*)	93.3 (*4,658*)	93.0 (*1,024*)	98.0 (*6,341*)	98.3 (*2,826*)	97.5 (*2,812*)	96.6 (*207*)
	R Age: >65	94.0 (*6,038*)	93.4 (*4,464*)	91.3 (*4,262*)	90.3 (*1,588*)	97.1 (*3,189*)	96.1 (*1,872*)	96.1 (*1,079*)	96.2 (*523*)
% 1-year patient and graft survival	R Age: 18-55	95.1	93.1	90.6	85.3	97.2	96.9	96.9	93.6
	R Age: 55-65	94.1	90.8	88.5	86.1	96.97	96.67	95.73	96.62
	R Age: >65	92.0	89.0	86.3	85.3	96.1	94.8	94.0	93.5
% with eGFR-1 > 90 mL/min	R Age: 18-55	18.8	7.2	3.1	0.7	17.7	7.2	4.8	3.2
	R Age: 55-65	14.3	5.2	3.3	1.1	11.3	4.3	2.1	3.6
	R Age: >65	10.1	4.0	1.7	1.2	7.5	2.8	1.3	0.4
% with eGFR-1 of 60-90 mL/min	R Age: 18-55	46.9	33.6	24.5	13.8	52.3	42.8	35.8	21.1
	R Age: 55-65	47.4	31.7	23.4	15.4	52.8	38.6	28.3	18.3

	**R Age:** >65	48.6	32.2	23.4	15.3	51.1	40.6	26.8	23.2
% with eGFR-1 of 45-60 mL/min	**R Age:** 18-55	21.7	31.5	32.9	27.3	21.8	33.2	35.0	43.2
	**R Age:** 55-65	24.7	32.0	30.4	29.1	24.9	37.8	38.9	39.6
	**R Age:** >65	25.5	30.9	31.3	30.3	29.7	37.4	39.6	36.3
% with eGFR-1 of 30-45 mL/min	**R Age:** 18-55	9.4	20.4	27.6	38.5	6.6	14.0	20.2	26.8
	**R Age:** 55-65	10.8	23.9	31.1	38.3	9.2	16.4	25.7	31.0
	**R Age:** >65	12.4	25.0	31.0	37.3	10.1	15.9	27.0	31.3
% with eGFR-1 < 30 mL/min	**R Age:** 18-55	3.2	7.3	11.9	19.7	1.7	2.7	4.2	5.8
	**R Age:** 55-65	2.8	7.2	11.9	16.1	1.9	2.9	5.1	7.6
	**R Age:** >65	3.5	7.9	13.1	15.9	1.6	3.3	5.2	8.8

Supplemental Table 2: Donor and Recipient characteristics and outcomes, stratified by KDPI and LKDPI. Increasing recipient death/graft failures with rising KDPI is reduced, but not eliminated through LKDPI stratification.

	**DDKT: 73,312 (for which KDPI can be calculated)**				**LDKT: 46,044 (for which LKDPI can be calculated)**			
**KDPI/LKDPI Range**	**0-0.5**	**0.5-0.75**	**0.75-0.9**	**0.9-1**	**< 15**	**15-35**	**35-60**	**60+**
**% Not utilized**	5.8	19.8	36.8	58.4	NA	NA	NA	NA
**Sample Size N**	41,719	19,238	8,699	3,656	26,294	13,310	5,580	860
**Donor Characteristics**								
Age: Median (IQR[a]), Mean	33 (24-42), 33.5	51 (46-55), 50.3	58 (53-62), 57.3	64 (59-68), 63.3	38 (30-46), 38.2	45 (36-53), 44.1	53 (42-60), 50.5	62 (55-66), 58.9
BMI: Median (IQR), Mean	26.4 (23.3-30.5), 27.5	27.8 (24.2-32.4), 28.9	27.8 (24.2-32.4), 28.9	27.5 (23.8-32.3), 28.7	26.1 (23.4-29.0), 26.3	27.3 (24.3-30.4), 27.5	27.9 (24.8-31.0), 28.1	28.2 (24.9-31.2), 28.4
% Female	33	48	52	57	52	72	76	78
% Caucasian	73	70.9	62.2	48	69.5	69.1	62.1	63.1
% African-American	8.7	13.1	22.7	37	5.5	17.6	29.4	31.5
% Hispanic	15	11.9	10.8	10	18.7	9.4	6	2.9
% Asian	1.8	3	3.2	4.1	4.8	2.5	1.5	1.6
% Other races	1.5	1.1	1.1	0.9	1.5	1.4	1	0.9
**Recipient Characteristics**								
Age: Median (IQR), Mean	53 (42-61), 51.3	57 (48-64), 55.4	61 (54-67), 59.8	64 (57-69), 62.6	48 (36-58), 47.3	50 (39-59), 49.0	54 (42-62), 51.5	60 (49-66), 56.7

BMI: Median (IQR), Mean	28.2 (24.4-32.2), 28.5	28.2 (24.7-32.2), 28.6	28 (24.6-31.9), 28.4	27.7 (24.4-31.5), 28.1	26.5 (23.3-30.4), 27.0	28.2 (24.4-32.5), 28.6	29.1 (25.0-33.4), 29.4	30.0 (25.6-34.0), 30.11
% Female	39.5	38.3	36.7	35.4	34.3	43.5	41.6	39.9
% Caucasian	43.5	42	41.9	40.6	66.4	65.9	58.7	61.1
% African-American	32.6	34.6	35.6	37.5	7.9	19.5	30.3	30.8
% Hispanic	16	14.5	13.5	12.8	18.6	9.8	6.6	4.2
% Asian	5.7	6.9	7.2	7.6	5.6	3.4	2.7	2.7
% Other races	2.2	2	1.8	1.5	1.5	1.4	1.7	1.2
**Transplant Characteristics**								
% with 3 or fewer HLA mismatches	29.4	23.3	15.8	13.4	63.6	45	34.3	22.9
% with delayed graft function	22.4	30.8	32.2	32.7	3.0	3.7	4.6	6.4
CIT: Median (IQR), Mean (hours)	16.1 (11-22), 17.4	17 (11.7-23), 18.1	17.2 (12-23.5), 18.6	18 (13-24), 19.2	1 (0.7-2), 2.1	1 (0.72-2), 2.1	1 (0.7-2), 2.3	1 (0.64-2), 2.2
% Biologically related	NA	NA	NA	NA	58.27	51.03	48	41.75
**Transplant Outcomes and eGFR-1**								
1-year patient and graft survival (%)	94.5	91.1	87	84.1	97.3	96.3	95.4	93.1
% with eGFR-1 > 90 mL/min	15.5	4.6	2.6	1.3	13.7	7.9	5.5	4.2
% with eGFR-1 of 60-90 mL/min	46.8	31.1	20.3	15.3	51.1	42.3	33.8	22.7

% with eGFR-1 of 45-60 mL/min	23.9	32.0	31.6	27.3	25.3	32.0	35.5	33.7
% with eGFR-1 of 30-45 mL/min	10.7	24.2	31.5	37.3	8.3	14.6	20.4	30.9
% with eGFR-1 < 30 mL/min	3.0	8.0	14.0	18.8	1.6	3.2	4.8	8.5

[a]IQR = Interquartile range

Supplemental Figure:

1. **Supp Fig 1. US waitlist (WL) composition at beginning and end of data analysis (gender, ethnicity, age) contrasted to DDKT and LDKT recipients during the analysis period:** The demographics of the kidney waitlist changed slightly over the decade, but the recipients of DD and LDKT differed from the WL.

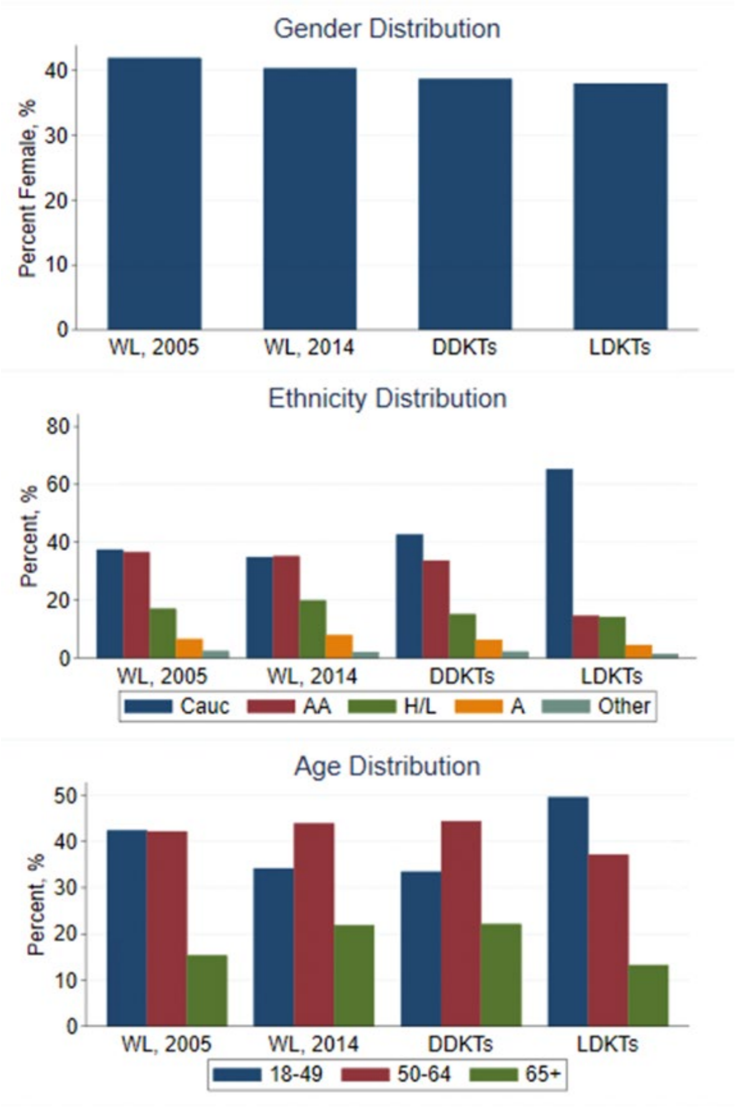

Supplement: Supplementary file 1 [file Datasheet1.pdf]
